# Case Cards: A Novel Learner-Centered Initiative to Improve Resident Education in the Pediatric Emergency Room

**DOI:** 10.7759/cureus.36944

**Published:** 2023-03-31

**Authors:** Elise Perlman, Selin Sagalowsky

**Affiliations:** 1 Pediatric Emergency Medicine, Cohen Children's Medical Center, New York, USA; 2 Pediatric Emergency Medicine, NYU (New York University) Langone Health, New York, USA

**Keywords:** pediatrics, pediatric emergency medicine, case cards, resident education, pediatric emergency medicine rotation, case-based teaching, learner-centered teaching

## Abstract

Introduction: The pediatric emergency department (PED) provides a wealth of learning opportunities for residents. However, delivering dedicated education can be a significant challenge due to considerable variability in day-to-day schedules, volume, cases, time, and resource availability. Case-based and learner-centered teaching models are well suited for ambulatory settings like the emergency department. Using the Kern model, we designed an educational intervention titled "Case Cards" to facilitate active learning conversations in pediatric emergency medicine (PEM). Our goal was to improve clinical teaching in the PED to demonstrate self-reported satisfaction, knowledge acquisition, confidence, and commitment among residents rotating in this fast-paced, challenging clinical environment.

Methods: After general and targeted needs assessments, we developed a compendium of 30 high-yield case cards to facilitate case-based learning conversations between learners and preceptors. Case topics mirror the American Board of Pediatrics Content Outline of Emergent Conditions. The "Learner Card" presents a PEM case for the learner to read and hold, while the "Teacher Card" contains evidence-based teaching prompts following established learner-centered clinical teaching models to guide and facilitate the case. Post-implementation surveys queried Kirkpatrick New World educational outcomes in a retrospective format.

Results: We collected data from 24 pediatric and emergency medicine residents between July 2021 and January 2022. All respondents agreed or strongly agreed that case cards are enjoyable, educational, applicable to clinical practice, enhanced confidence, and would be recommended to others.

Conclusion: Case cards for learner-centered teaching in the pediatric emergency setting are well-received and demonstrate resident self-reported satisfaction, knowledge, and confidence in core PEM conditions. Having established and readily available teaching topics, such as case cards, can enhance the clinical experience in the PED and other challenging settings and augment clinical exposure to core content. Educators may wish to expand and explore evolving technologies to facilitate learner-centered clinical teaching.

## Introduction

Despite a wealth of patient care and learning opportunities, resident education in the pediatric emergency department (PED) setting may be challenging due to variability in cases, volume, acuity, time, and resources [1]. For these reasons, learners may not be afforded the opportunity or exposure to the breadth of pathology comprising the core and required content. Clinical experiences can be augmented by effective on-shift teaching but are often limited due to competing demands and responsibilities in the PED. Furthermore, the quality of teaching depends on individual faculty member comfort and knowledge of specific topics and their teaching skills [1-4]. Structured preceptor models, including real-time resources, provide faculty with frameworks for case-based teaching in a timely and learner-centered fashion and have been shown to enhance teaching performance, collaborative learning, and critical thinking [5-8]. These models capitalize on cognitivist, constructivist, and behaviorist theories of adult learning in practical ways [9]. Recognizing these complex factors and functional barriers, our goal was to improve clinical teaching in the PED to demonstrate self-reported satisfaction, knowledge acquisition, confidence, and commitment among residents rotating in this clinical environment. We utilized the Kern model to develop, implement, and evaluate a new resource for learner-centered conversations utilizing printed pediatric emergency medicine (PEM) "Case Cards" [10].

## Materials and methods

During the 2018-2019 academic year, we conducted generalized and targeted needs assessments of resident learners in our PED that comprised reviewing institutional rotation evaluations from pediatric and emergency medicine residents, compiling relevant program evaluation data from the Accreditation Council for Graduate Medical Education (ACGME) annual residency program surveys, and implementing a targeted survey of pediatric residents’ educational experiences and perceived needs in PEM. These data identified resident discomfort with core PEM knowledge and skills and identified a need for dedicated clinical teaching. We applied the results of our needs assessment to devise the innovation of case cards to facilitate case-based learning conversations between learners and instructors.

Case cards comprise two components: a "Learner Card" and a "Teacher Card" (Appendix). The learner card presents a core PEM case, mirroring content from the American Board of Pediatrics Content Outline of Emergent Conditions [11]. Between April and August 2021, we developed a compendium of 30 high-yield case cards covering various topics: cardiac, respiratory, head and neck, gastrointestinal, ophthalmologic, endocrinologic, neurologic, hematologic, orthopedic, and traumatic domains. Case cards may be used in reference to a real-time clinical encounter (i.e., when a patient presents with the chief complaint listed) or separately for case-based teaching during a spare moment. They may also be used to facilitate independent learning and inquiry by residents and medical students. The teacher card contains evidence-based teaching prompts for facilitators to guide learners through the case, focusing on appropriate history, prioritized differential diagnoses, management plans, and clinical pearls, in line with established learner-centered clinical teaching models, such as One-Minute Clinical Preceptor, SNAPPS (summarize, narrow, analyze, probe, plan, and select), and activated demonstration [8]. Further asynchronous and self-directed learning is facilitated by a quick response (QR) code linking learners to Free Open Access Medical Education (FOAMed) resources, including blogs, podcasts, and videos for further reading on the topic. Case cards may take 5-10 minutes to facilitate depending on the availability of time, the context, and the level of discussion.

Printed case cards were readily available at the computer workstation and used at the discretion of the team and workflow. Resident surveys were distributed at the end of each four-week rotation, which queried Kirkpatrick New World educational outcomes of self-reported satisfaction, knowledge acquisition, confidence, and commitment in a retrospective fashion. These questions were enfolded into a larger survey about the overall PEM rotation.

## Results

We collected data from 41 pediatric (post-graduate year (PGY) 1-3) and emergency medicine residents (PGY1-2) between July 2021 and January 2022 (Table 1). Twenty-four residents answered questions specific to the case cards with 100% (n = 24) reporting improvements in all measured outcomes (Table 2). Qualitative comments highlighted ease, feasibility, and appreciation for self-directed learning (e.g., “They were easy to use and fast, useful knowledge”; “Easy to look through during downtime”; “Well laid out, great for use during less busy times”; “Being able to see a brief description of a case and delve into it on my own out of my own interest!”; and “Good thought exercises and helped include some structured learning in the day”).

**Table 1:** Total pediatric and emergency medicine residents surveyed PGY: post-graduate year.

Residents surveyed	N (%)
Emergency medicine	N = 7
PGY1	4 (9.8%)
PGY2	3 (7.3%)
Pediatrics	N = 33
PGY1	14 (34.1%)
PGY2	12 (29.3%)
PGY3	8 (19.5%)

**Table 2:** Combined resident survey responses

Survey responses		
	Agree, N (%)	Strongly agree, N (%)
Satisfaction: “They were enjoyable”	19 (80%)	5 (20%)
Knowledge acquisition: “I learned clinical pearls”	18 (78%)	5 (22%)
Confidence: “From Case Cards, I feel more confident taking care of pediatric patients”	18 (78%)	5 (22%)
Commitment: “I will incorporate what I learned into my practice”	18 (78%)	5 (20%)
I would recommend “Case Cards” to others	17 (74%)	6 (26%)

## Discussion

While numerous structured models exist to facilitate clinical teaching in emergency departments, these generally rely on clinical encounters and are subject to patient volume, acuity, breadth, and preceptor knowledge and skills [8]. Existing literature upholds that case-based learning promotes active discussion, creates a forum for inquiry, and strengthens relationships among participants while structured preceptor models and real-time resources provide faculty with the framework in which to do so [3,5-8]. Our intervention of case cards combines these two fundamental concepts to facilitate collaborative, targeted, and guided clinical teaching on-shift, with or without a clinical encounter. Importantly, case cards can be used to address the variability in volume, acuity, and breadth of pathology that occurs during any given rotation by providing learners with alternate means of exposure to core PEM content. Finally, in addition to case-based learning, each card has a QR code linking learners to related FOAMed resources to promote asynchronous and self-directed learning, which has been found to improve learner motivation and overall content retention [12,13]. In our implementation of case cards, we chose to use printed materials at the provider workstation rather than digital content, due to feedback regarding barriers such as computer workstation availability, time spent logging in, and desire for personalized group conversations.

Limitations

The breadth of our innovation was limited by time, as we were able to develop only 30 high-yield case cards rather than addressing all the conditions listed in the American Board of Pediatrics outline. Our evaluation did not query faculty perspectives on this teaching tool, nor did it specifically investigate the feasibility, though some of these data emerged in qualitative learner comments. We did not survey the use of each case card as they were used due to time, competing responsibilities, and the need for patient care. Our evaluation was conducted in a retrospective format and is subject to sampling, non-response, and recall bias.

## Conclusions

Case cards facilitate learner-centered teaching in the PED and may improve resident satisfaction, knowledge, and confidence in core conditions. Having established and readily available teaching topics with resources, such as case cards, can augment clinical exposures and improve knowledge acquisition in the PED and other challenging settings. Educators may wish to expand and explore evolving technologies to facilitate learner-centered clinical teaching.

## Figures and Tables

**Figure 1:**
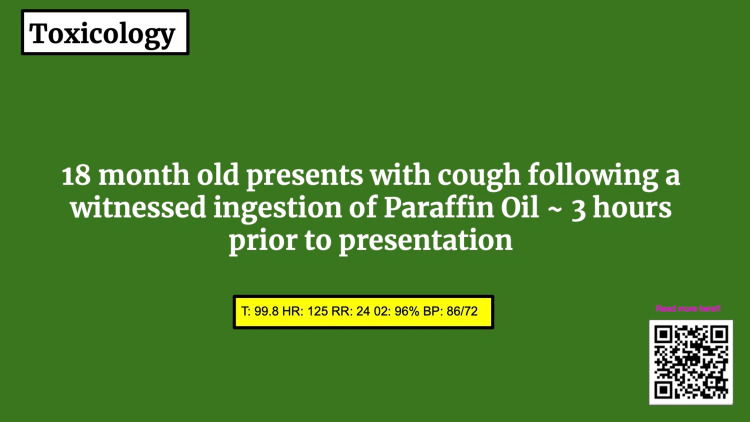
Example: learner card

**Figure 2:**
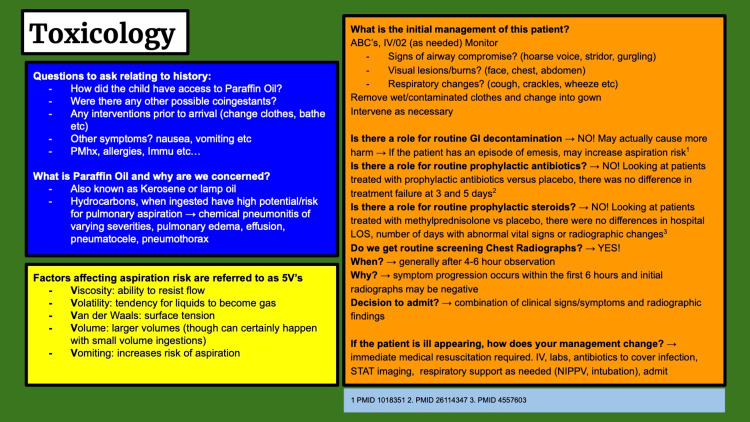
Example: teacher card

## Appendices

Case cards
